# Self-organizing peer coach groups to increase daily physical activity in community dwelling older adults

**DOI:** 10.1016/j.pmedr.2020.101181

**Published:** 2020-08-21

**Authors:** Paul van de Vijver, Frank Schalkwijk, Mattijs E Numans, Joris P.J. Slaets, David van Bodegom

**Affiliations:** a Leyden Academy on Vitality and Ageing, Rijnsburgerweg 10, 2333AA Leiden, The Netherlands; b Public Health and Primary Care, Leiden University Medical Center, Albinusdreef 2, 2300RC Leiden, The Netherlands; c University Center for Geriatric Medicine, University Medical Center Groningen, Hanzeplein 1, 9713GZ Groningen, The Netherlands

**Keywords:** Peer coaching, Physical activity, Older adults, Implementation, Replicability, Feasibility

## Abstract

Many older adults do not reach the recommended level of physical activity, despite many professional-delivered physical activity interventions. Here we study the implementation of a novel physical activity intervention for older adults that is self-sustainable (no financial support) and self-organizing (participants act as organizers) due to peer coaching. We implemented three groups and evaluated process and effect using participatory observations, questionnaires, six-minute walk tests and body composition measures from October 2016 to September 2018. The intervention was implemented by staff without experience in physical activity interventions. Facilitators were a motivated initiator and a non-professional atmosphere for participants to take ownership. Barriers were the absence of motivated participants to take ownership and insufficient participants to ensure the presence of participants at every exercise session. The groups exercised outside five days a week and were self-organizing after 114, 216 and 263 days. The initial investments were 170€ for sport equipment and 81–187 h. The groups reached 118 members and a retention of 86.4% in two years. The groups continue to exist at the time of writing and are self-sustainable. Quality of life increased 0.4 on a ten-point scale (95%CI 0.1–0.7; p = 0.02) and six-minute walk test results improved with 33 m (95%CI 18–48; p < 0.01) annually. Self-organizing peer coach groups for physical activity are feasible, have positive effects on health and require only a small investment at the start. It is a sustainable and potentially scalable intervention that could be a promising method to help many older adults age healthier.

## 1 Introduction

Daily physical activity is effective at preventing many age-related diseases such as diabetes and cardiovascular diseases and improving mobility and mental health (Vogel et al., 2009). In spite of these health benefits, approximately half of adults older than 60 worldwide do not meet the recommended level of physical activity (Hallal et al., 2012, Sun et al., 2013). Many interventions aiming to increase or intensify physical activity are proven effective in studies, but rarely reach practice (Hallal et al., 2012, Durlak and DuPre, 2008). Transferring effective programs into real world settings and maintaining them there is a complicated, long-term process depending on how well the program is implemented, and whether the program is sustainable (Durlak and DuPre, 2008). Thorough implementation increased effect sizes of intervention on average two to three times (Durlak and DuPre, 2008). However, over the past thirty years only 3% of physical activity studies focussed on implementation and dissemination (Milat et al., 2011). A study shows that the decision to adopt an intervention by policy makers is depending on data on effectiveness, reach and costs of operating at scale (Milat et al., 2014). To increase the likelihood of successful implementation and scale up of population physical activity interventions elsewhere, characterizing parameters of implementation setting, identifying stakeholder and identifying barriers and facilitators of interventions is necessary (Koorts et al., 2018).

Factors affecting the implementation process are the intervention (e.g. compatibility, adaptability), the provider of the intervention (e.g. perceived need, perceived benefits, self-efficacy, skills), the organization (e.g. innovation, work climate, communication) and the community (e.g. politics, funding, policy) (Durlak and DuPre, 2008, Koorts et al., 2018, McKay et al., 2017). Of these factors, one of the main barriers for successful sustainable implementation at scale are costs and scarcity of professionals to deliver these intervention (De Ponte et al., 2014, Hall et al., 2010, Van der Bij et al., 2002). Physical activity interventions for older adults can cost 1000 dollar or more per participant. (Groessl et al., 2009) Peer coaching, in which participants act as coach and organizer is a novel method without these barriers (Matz-Costa et al., 2018, Thom et al., 2013, Sokol and Fisher, 2016). A systematic review showed substantial health benefits for a variety of peer interventions, although not in all (Ramchand et al., 2017). Further research showed that peer-based interventions benefit if they are (co-) developed by peers (Raja et al., 2008). Moreover, the use of peers increased the long term maintenance of physical activity interventions (Buman et al., 2011). Another study suggested however that peers need regular supervision (Raja et al., 2008). In a previous study we described the Vitality Club, a self-organizing physical activity intervention that exercises outside five days a week and sustainably activated a group of 70 older adults for over 6 years. Participants attended on average two and a half days a week and perceived both subjective and objective improvements in health and well-being (Van de Vijver et al., 2018). The first Vitality Club started in 2010 by older adults themselves and at the time of writing continues to exist with over 150 members without any professionals or financial support involved (Van de Vijver et al., 2018). This local intervention managed to increase physical activity in large numbers of older adults in a sustainable way, but to improve population health it must be delivered at scale (McKay et al., 2017). The initial Vitality Club was created by already motivated older adults who are still organizing it, and the question remains if it is feasible for healthcare professionals to implement a similar self-organizing physical activity intervention in a real-world setting that eventually will be sustained and organised by participants themselves.

Therefore, we studied the implementation of a peer coach physical activity intervention for older adults in a real-world setting and evaluate factors affecting implementation, effectiveness on health and well-being, costs, implementation setting and stakeholders. We initiated three new peer coach groups in different neighbourhoods in Leiden. This implementation study is necessary for future dissemination of the intervention. Also, the results can have important implications for the wider use of peer coaching in health promotion or disease management, where peer coaching could also be a novel method of intervention delivery for its promising sustainable and low-cost characteristics.

## 2 Methods

### 2.1 Study design

The study took place from October 2016 till September 2018. We implemented three peer coach groups in three neighbourhoods in Leiden, a medium sized town in The Netherlands with 120,000 inhabitants. Number of inhabitants in the neighbourhoods ranged from 11,149 to 21,467. The proportion of inhabitants older than 65 ranged from 11.8 to 18.1% in the neighbourhoods. Leiden is a city in a densely populated area with a different demographics than the original setting, which was a large village in one of the more rural areas of the Netherlands. Social cohesion tends to weaker in cities and neighbours do not know each other. This makes it an interesting location to implement an intervention that is dependent on social cohesion.

The following practical implementation steps were conducted. Firstly, we identified and contacted public places suitable for exercise in the neighbourhood, with a possibility to store a small box with sport equipment. Secondly, we supplied sport equipment for the first ten participants, including 1 kg dumbbells, mini soccer balls and elastic bands. The costs per set were around 17 EUR. Thirdly, we recruited participants using a less formal and low budget recruitment strategy with flyers, free advertorials in local newspapers and neighbourhood associations. There was no formal age requirement, but advertisement mentioned that the exercise intensity was aimed at people aged older than 55. There was no required baseline level of physical activity, but participants had to come to the Vitality Club independently. Finally, peer coaches were recruited from the participants as the groups grew. To empower participants to take ownership we created an atmosphere where participants were free in the way they organised the exercise sessions. We always stressed that it was not a professional intervention, but a group of neighbourhood peers exercising together. All participants that volunteered to take the role of peer coach could become a peer coach, and they distributed the exercise sessions among the peer coaches. During the implementation phase, exercise sessions for which no peer coach was available were led by the researchers until a peer coach was recruited. The switch from staff to peer coach was done during one session in which staff and peer coach together led the session. The groups gathered 5 days a week from 09.00 to 10.00 in the morning. The exercises were free format, but mostly of moderate intensity. Most coaches focused on cardiovascular exercises, muscle strengthening exercises and exercises for stability and flexibility. However, because of the free format, each coach could choose any kind of exercise. Peer coaches received only a non-formal training through observing sessions led by researchers and other peer coaches. Researchers were recently graduated medical doctors who did not received special training to guide exercise sessions. Peers only received supervision when requested. To make the peer groups fully self-sustainable (financially independent) a voluntary fee of 1 EUR per week was installed. The peer groups themselves managed the funds and used it to cover expenses and buy additional sports equipment as the groups grew. Participants were made aware that attendance was at their own risk and health issues would be handled similar as all outside injuries would, minor injuries at home or the general practitioner, major issues by calling the emergency number.

We made participatory observations and conducted semi-structured interviews to identify facilitators and barriers for the implementation of the peer coach group. All participants also received a questionnaire at baseline and every four months to assess personal facilitators and barriers. We recorded daily presence and conducted a six-minute walk test and bioelectrical impedance analysis (BIA) at baseline and every four months. Participants that did not participate in the exercise group for 90 days were considered drop-outs. For the BIA, the OMRON BF511 body composition monitor was used (Omron Healthcare Ltd., Kyoto, Japan). The questionnaire was similar to the questionnaire we used before and included questions on demographic characteristics, physical activity frequency and quality of life (Supplementary S1) (Van de Vijver et al., 2018). At the fifth participation we requested informed consent for participation in the study since we did not want to interrupt the real-world setting and wanted people to be able to join the club without participating in the study. After people joined the Vitality Club, consent was always asked to be included in the study. All study participants in the study provided written informed consent before assessment started.

### 2.2 Statistics

We analysed the intraindividual change over time using a Linear Mixed Model. This model separates within-person change and between-person differences in outcomes over time (Shek and Ma, 2011). The model showed that 79% of the variance in outcome is attributed to differences between individuals, warranting the use of this model. The model had a slightly better fit using a quadratic regression, but the change after one year was similar in the quadratic and linear regression. Therefore, we used a linear regression because the estimates are more easily interpreted. The model was adjusted for sex, age at baseline, height and weight with unstructured covariance and intercept and time as a random variable. The analysis of body composition change was not adjusted for weight. Predicted six-minute walk test results were calculated using the regression of Troosters et al. (1999) and were used as reference values (Troosters et al., 1999). Statistical analyses are performed with IBM SPSS Statistics for Macintosh, Version 25.0, Armonk, NY: IBM Corp.

## 3 Results

The practical implementation strategy was flexible in different settings and easy to conduct by staff without experience in physical activity interventions. This implementation strategy resulted in three peer coach groups with the essential elements of the intervention (exercising, self-organizing and sustainable). The stakeholders of the intervention are the initiator, the participants, the peer coaches and the owner of the exercise/storage location. Implementation facilitators are collaborative stakeholders, a well-informed and motivated initiator and a non-professional atmosphere for participants to take the role of peer coach. The barriers are the absence of motivated participants willing to take over the exercise sessions and insufficient number of participants to ensure the presence of participants at every exercise session. To overcome these barriers the peer coach group needs to be of a sufficient size, which could take time due to the less formal recruitment strategy. An initiator needs to stay motivated until the group reaches a critical mass.

Table 1 shows baseline characteristics of participants and peer coaches. In total 132 people were asked for informed consent, 118 people provided informed consent for the study, resulting in an inclusion rate of 89%. Average age was 66.9 (SD 6.4) year, 74% of participants were female, 73% was retired and 33% was living alone. Of all participants 56% had a high educational level. Monthly disposable income was under €1000 for 12% of participants, between €1000 and €3000 for 63% and more than €3000 for 25% of participants.

**Table 1:** Baseline characteristics of study participants.

	Peer coaches		All participants	
Number	13		118	
Age in years, mean (*SD)*	68.8	−2.6	66.7	−6.6
Women, n (%)	9	−69%	87	−74%
Retired, n (%)	12	−92%	82	−73%
Educational level, n (%)^a^				
Low	1	−8%	22	−20%
Middle	3	−23%	27	−24%
High	9	−69%	62	−56%
Disposable income, n (%)^b^				
<€1000	1	−13%	9	−12%
€1000–€3000	4	−50%	46	−63%
>€3000	3	−37%	18	−25%
Living alone, n (%)	1	−8%	37	−33%

Total number of participants varies due to missing data.

a Low educational level is International Standard Classification of Education (ISCED) 0–2. Middle educational level is ISCED 3–4. High educational level is ISCED 5–8.

b Disposable income per household is total income per household minus taxes and social fees.

**Fig. 1:**
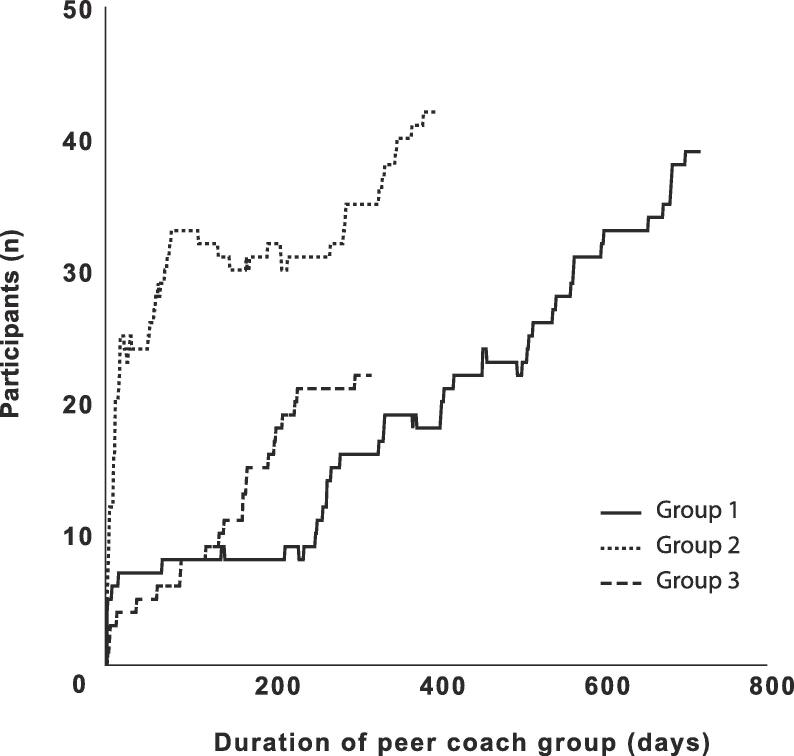
Growth of the peer coach groups. The number of participants of the peer coach group over time October 2016 to September 2018. Group 1 started in October 2016, group 2 started in August 2017 and group 3 started in November 2017. Only participants that provided informed consent are included. Participants that did not participate in the last three months were considered a drop-out.

At the start, 47% of participants were recruited by an article in the local newspaper, 15% via word of mouth and 8% via a flyer delivered at home. Throughout the study period, most participants were recruited via word of mouth (39%). Fig. 1 shows the number of participants of the peer coach groups over time. We identified several facilitators and barriers for joining and remaining with the peer coach group from our semi structured interviews and questionnaires. The main facilitators were exercising outside, exercising early morning, the feeling of social obligation to the group, variations in exercises due to different peer coaches, low participation fee, the fact that no formal enrolment was required and that the participants were of similar age. Personal barriers to join were the distress of joining a new group of people and not being sure of having the adequate physical fitness to participate, a lack of motivation and the early timing of the sessions. Motivational facilitators for the peer coaches were the satisfaction of helping peers live healthier, being in charge of the intervention and being able to adjust exercises to personal preferences. A barrier to becoming a peer coach was the perceived stress of leading a group.

The peer coach groups became completely self-organizing 114 (group 1), 216 (group 2) and 263 (group 3) days after the start. At the time of writing, the groups exercise five times a week and are still growing without any supervision from the researchers for 1156 (group 1), 733 (group 2) and 610 (group 3) days after becoming completely self-organizing. Until the groups became self-organizing, the researcher invested one hour a day to lead the group exercises. The time investment of the researcher gradually declined when a peer coach was recruited from the participants for one or several weekdays. Total investment per group was 170€ for sport equipment and 81 (group 1), 154 (group 2) and 187 (group 3) hours. On average participants attended the peer group 1.5 (SD 0.8) days per week. The median number of participants on a regular day was 6 (range 0–18). On a weekly basis the median number of unique participants was 28 (range 11–56). During the study period 16 participants were absent for more than 90 days and were considered dropouts, resulting in a retention of 86.4% during the study period of 2 years. The attrition rate was 0.19 per person-year of observation (total of 82.1 person-years of observation). Only 3 participants returned after an absence of 90 days, but were still considered drop-outs. There were 4 participants that were absent for more than 60 days, but <90 days when the study ended and were not considered drop-outs. There were between 3 and 5 peer coaches per group at any time. One peer coach had to stop due to illness. It was easier to replace an absent peer coach (e.g. illness) when more peer coaches were available. Some participants preferred the teaching style of a certain peer coach and selectively participated in the peer group on days when that peer coach was coaching.

Table 2 shows baseline characteristics and yearly change of health and well-being. We had 244 measurements for the 118 participants, an average of 2.1 measurements per participant. Participants reported an annual increase of 1.8 days per week of physical activity >30 min (95%CI 1.0–2.5; p < 0.01). Self-reported quality of life improved with 0.4 points on a ten-point scale per year (95%CI 0.1–0.7; p = 0.02). Six-minute walk test results increased with 33 m per year (95%CI 18–48; p < 0.01). Participants lost 1.4 kg annually (95%CI –2.6 to −0.3; p = 0.01). BMI reduced with 0.5 points (95%CI −0.9 to −0.1; p = 0.02). In the general population six-minute walk test results decline on average 5 m a year due to ageing (Troosters et al., 1999). The group started with a 6MWT result close to the predicted result for their respective age, gender and height, 99% of predicted and improved with 6.2 percent points (95%CI 3.8–8.5; p < 0.01) after one year. Participants improved independent of their baseline results.

**Table 2:** Baseline characteristics and yearly change of health and well-being.

	Baseline (SD)		Yearly change (SE)^a^		p-value
Self-reported days per week >30 min PA	2.5	(1.8)	+1.8	(0.3)	<0.01
Quality of life (1–10)	7.7	(1.0)	+0.4	(0.1)	0.02
Observed 6MWT result in meters	607	(71)	+32.9	(7.3)	<0.01
Predicted 6MWT result in meters^b^	614	(55)	−5.3	(0.0)	
Observed/predicted 6MWT * 100%	99.3	(12.1)	+6.2	(1.2)	<0.01
Body weight (kg)	74.5	(12.6)	−1.4	(0.6)	0.01
BMI (kg/m^2^)	25.9	(3.6)	−0.5	(0.2)	0.02
Fat percentage	32.8	(9.5)	−1.0	(0.6)	0.08
Muscle percentage	28.9	(4.5)	−0.1	(0.5)	0.89
Visceral fat score (1–20)	9.1	(3.4)	+0.4	(0.2)	0.82

PA = physical activity, 6MWT = six-minute walk test, BMI = body mass index.

a Estimates derived from a linear mixed model adjusted for sex, age at baseline, height and weight, using 244 measurement moments from 118 participants. The analysis of body composition change was not adjusted for weight.

b Predicted six-minute walk test distance calculated from the equation of Troosters et al. (1999). Data collected from October 2016 to September 2018.

We also analysed a dose-response relation. Participants improved 0.3 m per participated session (95%CI 0.1–0.5; p = 0.01). No serious injuries were reported during the study period. Six participants (4%) reported a minor sport related injury. There was full recovery of all injuries.

## 4 Discussion

This study shows a feasible implementation strategy to initiate peer groups as a daily physical activity intervention for community dwelling older adults. The peer coach groups became completely self-organizing after the initial researcher-led period and at the time of writing continued to exist and grow.

The implementation of peer coach groups comprised of practical steps that can be performed in a lot of different settings by different people. This study has been performed in the cultural background of West-Europe, but similar initiatives can be seen in South-America and Asia (González Ramos et al., 2013). However, depending on cultural etiquette, changes need to be made to the implementation strategy. There are several recommendations for others who want to implement this intervention. Key factors are the number of participants and empowerment of participants to take ownership. The first factor can be achieved with the same less formal recruitment strategy used in this study, but this method takes considerable time and initiators should be made aware of this from the start. The groups mostly grew through word of mouth, which does not require action but is hard to predict. The second factor depends on the atmosphere created by the initiator. We learned that the participants must be informed from the start that it will be a peer-led intervention. It must be stressed that almost all participants are able to lead an exercise session. Otherwise, participants will not feel empowered to take ownership. This study showed that when a group does take ownership, it is resilient and can exist for a long period without any supervision.

The sustainability of peer coach groups for physical activity in older adults can be understood from several theoretical perspectives. Firstly, peer coaches bring several advantages which are derived from social support, experiential knowledge, helper-therapy principle, social learning theory and social comparison theory (Solomon, 2004). Secondly, the groups are self-organizing and self-sustainable increasing self-efficacy and internal locus of control, which in turn are strong predictors of adherence (McAuley et al., 2003). Lastly, social interaction is an essential part of this physical activity intervention, which has additional benefits on adherence and on the well-being of participants (McAuley et al., 2000, Smith et al., 2017).

From a practical perspective, as there are no paid professionals or costly sporting accommodations required, there are no fixed expenses for the intervention. Moreover, because the intervention is not dependent on scarce and costly professionals and can be set up anywhere in the public space, it is potentially scalable.

A limitation is that the intervention mostly attracts healthy active older adults with a high socioeconomic background. However, research on the first peer coach group that existed for over 6 years at the time showed that 50% of participants had low educational status (Van de Vijver et al., 2018). Also, healthy participants will become more frail due to ageing in the following years. We did not record race in this study. We are currently conducting a study with peer coach group for older adults with a migratory background in which we also include race. Another limitation is that this study tests the feasibility of only three peer coach groups in Leiden. To make the results more generally applicable we used neighbourhoods with three different average socioeconomic statuses to set up the peer coach groups. However, they are all situated in the same city thus being relatively similar. Additionally, due to small number of peer groups we were unable to formally compare different implementation processes to distinguish the optimal strategy. Finally, the effects of the peer coach intervention on its participants were not compared with a control group and causal relations must be taken cautiously. However, daily physical activity of participants increased while daily physical activity in older adults in general decline with increasing age (Sun et al., 2013).

The unique aspect of this form of intervention is the absence of a professional. A common argument against peer coaching is that it is inferior and unsafe to use peers instead of professionals to lead the intervention. A systematic review however, showed that peer-based physical activity interventions were equally effective as interventions led by professionals for increasing physical activity (Ginis et al., 2013). Additionally, no study have shown a difference in safety between interventions delivered by peers or professionals (Castro et al., 2011). Furthermore, in a randomized controlled trial comparing peers to professionals in a physical activity intervention, peer coaches were found to be equally or superior to professionals in levels of intervention implementation (Buman et al., 2011).

Peer support is already used widely in disease prevention in diabetes, cardiovascular disease and cancer patient groups, to quit smoking or to stay sober in the Alcoholics Anonymous. However, most physical activity interventions still use a professional as primary leader of the intervention. We like to stress the possibility of increasing the role of the peers in physical activity intervention. This does not come with a decrease in quality and brings the big benefits of peer support, low costs, scalability and could reach a part of the population currently not reached by classic interventions.

## 5 Conclusion

It is feasible for healthcare professionals to implement a peer coach physical activity intervention for older adults that eventually is self-organizing and self-sustainable. A small investment of 170€ and 81–187 h is needed to create a group that is self-supporting after 114–263 days. After this initial investment the groups are self-organizing and self-supporting and resilient for a long period. This novel form of intervention delivery could be a promising alternative model to curtain increasing healthcare expenditure and could potentially help large numbers of older adults age healthier.

## CRediT authorship contribution statement

**Paul van de Vijver:** Conceptualization, Data curation, Formal analysis, Funding acquisition, Investigation, Methodology, Supervision, Validation, Visualization, Writing - original draft. **Frank Schalkwijk:** Conceptualization, Investigation, Methodology. **Mattijs E Numans:** Conceptualization, Supervision. **Joris P.J. Slaets:** Conceptualization, Supervision. **David van Bodegom:** Conceptualization, Funding acquisition, Methodology, Project administration, Supervision, Validation.
