# Supplementary material for: Self-organizing peer coach groups to increase daily physical activity in community dwelling older adults
Source: Prev Med Rep. 2020 Aug 21;20:101181. doi: 10.1016/j.pmedr.2020.101181 (PMC7567038; doi:10.1016/j.pmedr.2020.101181)
Supplement: Supplementary data 1 [file mmc1.docx]

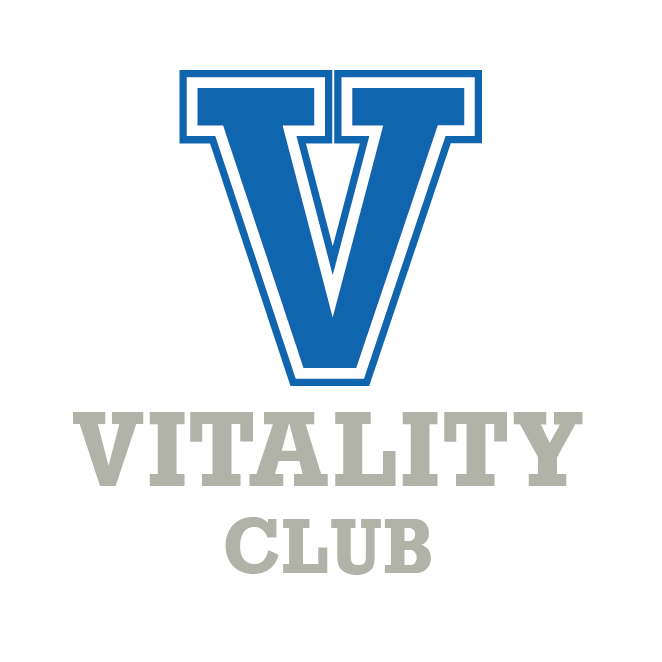
*This survey was used in the study ‘Self-organizing peer coach groups to increase daily physical activity in community dwelling older adults’ and was originally in Dutch. The survey is translated to English for publication purpose, not research purpose.*

# Survey

## General health

### In general, would you say your health is:

| Excellent | 🞎 |
| --- | --- |
| Very Good | 🞎 |
| Good | 🞎 |
| Fair | 🞎 |
| Poor | 🞎 |

### Your quality of life as a whole is:

| Very bad | | | |  | |  | |  | |  | Very good | | | | |
| --- | --- | --- | --- | --- | --- | --- | --- | --- | --- | --- | --- | --- | --- | --- | --- |
|  | | | |  | |  | |  | |  | | |  | | |
| 1 | 2 | 3 | 4 | | 5 | | 6 | | 7 | | | 8 | | 9 | 10 |

## Motivations and barriers

The next question are about participating in the Vitality Club.

### How did you know about the existence of the Vitality Club?

………………………………………………………………………………………………………………………………………………………………………………………………………………………………………………………………………………………………………………………………………

### How many days did you know about the Vitality Club before participating for the first time?

……………………………………………………………………………………………………………

### What was the direct reason you joined the Vitality Club?

…………………………………………………………………………………………………………………………………………………………………………………………………………………………

### What do you hope to gain? (*multiple responses possible*)

| 🞎 Stay fit | 🞎 Meet new people |
| --- | --- |
| 🞎 Lose weight | 🞎 Have fun |
| 🞎 Be outside frequently | 🞎 Something else, ……………………… …………………………………………… |

### What was the reason you did not join the Vitality Club immediately?

…………………………………………………………………………………………………………………………………………………………………………………………………………………………

### Did you experience barriers to join the Vitality Club? If so, what were they?

………………………………………………………………………………………………………………………………………………………………………………………………………………………………………………………………………………………………………………………………………

### What aspects of the Vitality Club appealed to you?

………………………………………………………………………………………………………………………………………………………………………………………………………………………………………………………………………………………………………………………………………

### What do you think of the Vitality Club concept?

- Very good
- Good
- Bad
- Very bad

### The Vitality Club increases daily physical activity of people living in your neighbourhood?

| **Strongly disagree** | | | |  | |  | |  | |  | **Strongly agree** | | | | |
| --- | --- | --- | --- | --- | --- | --- | --- | --- | --- | --- | --- | --- | --- | --- | --- |
|  | | | |  | |  | |  | |  | | |  | | |
| 1 | 2 | 3 | 4 | | 5 | | 6 | | 7 | | | 8 | | 9 | 10 |

### How many member of the Vitality Club did you already know? Please state their names.

…………………………………………………………………………………………………………………………………………………………………………………………………………………………

### What do you think is ideally the maximum daily group size of the Vitality Club? Why?

…………………………………………………………………………………………………………………………………………………………………………………………………………………………

### Do you think you could be a peer coach?

- yes
- no

### Do you want to be a peer coach?

- yes
- no

### Which days do you prefer to exercise in the Vitality Club?

- Monday
- Tuesday
- Wednesday
- Thursday
- Friday

### How many times a week do you plan to exercise in the Vitality Club?

……………………………………………………………………………………………………………

### What is a reasonable amount to pay on a weekly basis for the Vitality Club?

I find €……………a week a reasonable price to pay for the Vitality Club.

## Current state

De next questions are to examine the current state on several aspects. The question will be repeated in four months.

|  |  |  | **On average the last four months** |
| --- | --- | --- | --- |
| a. | How many days a week do you exercise 30 minutes or more? |  | ___ |
| b. | How many social arrangements do you have a week? |  | ___ |
| c. | How many days were you ill? |  | ___ |
| d. | How many times did you visit the general practitioner? |  | ___ |
| e. | How many pills do you take daily? |  | ___ |
| f. | How would you rate your quality of sleep? (1-10) |  | ___ |
| g. | How would you rate your physical fitness? (1-10) |  | ___ |
| h. | How would you rate your knowledge of a healthy lifestyle? (1-10) |  | ___ |
| i. | How would you rate your quality of life? (1-10) |  | ___ |

## Personal information

All information will be stored anonymously. If you do not want to answer a question, you can skip it and go to the next.

| Name | ……………………………………………………….. |
| --- | --- |
| Height | ………………………………………………………..cm |
| Marital status | - Single, never married - Married - Divorced - Widowed |
| Household characteristics | - Living with partner, without children - Living with partner, with children - Living with partner and other people - Living without partner, with kids - Living without partner, with other people - Living alone |
| If you have a partner, is he a member of the Vitality Club? | - Yes, name partner:……………………………… - No - I have no partner |
| Living situation | - Living independently - Living with a private individual - Nursing home - Assisted living - Other |
| Educational level | - Lowest: *(ISCED 0, CBS classification)* - Low: *ISCED 1-2, CBS classification)* - Middle: *(ISCED 3-4, CBS classification)* - High: *(ISCED 5-8, CBS classification)* - Other:………………………..... |
| Disposable monthly income (total income per household minus taxes and social fees.) | - 0 - 500 euro - 500 - 1.000 euro - 1.000 - 1.500 euro - 1.500 - 2.000 euro - 2.000 - 2.500 euro - 2.500 - 3.000 euro - More than 3.000 euro |
| Are you currently employed? | - yes - no, I am 🞎 retired   🞎 unemployed  🞎 other,…………………. |
